# Sex differences in heart failure: an analytical cross-sectional study at Laquintinie Hospital, Douala, Cameroon, 2021-2024

**DOI:** 10.11604/pamj.2026.53.34.48976

**Published:** 2026-01-27

**Authors:** Djibrilla Siddikatou, Marie Solange Ndom, Valérie Ndobo, Mandeng Ma Linwa Edgar, Sidick Mouliom, Hermann Tsague, Raissa Tchounja Kamgang, Ali Abas, Ba Hamadou, Seck M’baye M’baye Salissou, Félicité Kamdem

**Affiliations:** 1 Faculty of Medicine and Pharmaceutical Sciences, University of Douala, Douala, Cameroon,; 2 Faculty of Medicine and Biomedical Sciences, University of Yaoundé I, Yaoundé, Cameroon,; 3 Faculty of Health Sciences, University of Buea, Buea, Cameroon,; 4 Cardiology Unit, Internal Medicine Department, Hôpital Laquintinie Douala, Douala, Cameroon,; 5 Faculty of Medicine and Biomedical Sciences, University of Garoua, Garoua, Cameroon,; 6 Faculty of Health Sciences, André Salifou University of Zinder, Zinder, Niger

**Keywords:** Heart failure, gender differences, atrial fibrillation, renal disease, Cameroon

## Abstract

**Introduction:**

heart failure (HF) is a major cause of mortality in sub-Saharan Africa. Limited data exist on sex-specific differences in HF presentation and outcomes. This study assessed sex-based variations in clinical profiles, aetiologies, and in-hospital outcomes among HF patients in Cameroon.

**Methods:**

we conducted an analytical cross-sectional study of patients admitted with clinical HF at Laquintinie Hospital, Douala (January 2021-December 2024). Patients without gender or outcome data were excluded. Sociodemographic, clinical, and treatment variables were compared by sex using appropriate statistical tests (p<0.05). Penalized logistic regression, validated via bootstrapping, modeled four outcomes: atrial fibrillation, ischemic cardiomyopathy, renin-angiotensin-aldosterone system (RAAS) inhibitor use, and hypertension. Missing data were imputed.

**Results:**

among 757 patients, 51.3% were female. Hypertension was present in 57.1%, more common in females (64.4% vs. 49.3%, p<0.001), with nearly equal known and new-onset cases. Females were older (median age 66 vs. 60 years, p<0.001) and had higher atrial fibrillation risk (AOR=3.64, 95% CI: 1.91-6.94). Males had more ischemic cardiomyopathy (AOR=0.48, p=0.035) and renal dysfunction (43.8% vs. 30.1%, p=0.003). Females more frequently received RAAS inhibitors/ARNI at discharge (33.5% vs. 26.3%, p=0.031). In-hospital mortality was 17.0%, with no sex difference (p=0.288).

**Conclusion:**

significant sex differences exist in HF presentation and treatment at Laquintinie. Strategies should include atrial fibrillation screening in females and renal monitoring in males. Findings support the need for sex-specific interventions and multicenter validation in similar settings. These cross-sectional associations highlight prevalence patterns but require prospective studies for causal insights.

## Introduction

Heart failure (HF) affects 55.5 million people globally, with a disproportionate burden in sub-Saharan Africa, where in-hospital mortality reaches 26.4% [1,2]. Sex differences in HF risk factors drive distinct phenotypes: females often present with hypertension-driven HF with preserved ejection fraction (HFpEF), while males exhibit ischemic-driven HF with reduced ejection fraction (HFrEF) [3].

Main risk factors for HF include coronary heart disease, hypertension, diabetes mellitus, a family history of heart disease, obesity, chronic pulmonary diseases, inflammation or chronic infection, metabolic diseases, and treatment with cardiotoxic agents (cocaine, anthracycline therapy in oncology, e.g., doxorubicin, trastuzumab in the treatment of breast cancer, etc.) [4]. Because the incidence of these risk factors may differ based on biological sex, many studies have explored the role of gender on incidence and outcomes of HF based on biological sex [3,5,6]. Acute coronary syndromes (ACS) have been reported to occur three to four times more often in men than in women aged below 60 years; however, mortality from ischemic heart disease is higher in women [3]. Peripartum cardiomyopathy, which occurs exclusively in women, is a life-threatening pathology [7], which may therefore disproportionately affect HF mortality in women. Endocrine disorders like hyperthyroidism and its associated thyroid cardiomyopathy, Takotsubo Cardiomyopathy, rheumatic heart disease, as well as comorbidities like diabetes mellitus have all been reported to occur more commonly in women [7-9].

In Cameroon, hypertension is a critical HF risk factor, with high prevalence and significant new-onset cases, particularly in females, underscoring gaps in primary care [10]. The sub-Saharan Africa Survey of Heart Failure (THESUS-HF, 2007-2010) reported higher atrial fibrillation in females and worse renal markers in males, but recent data are limited [11]. Resource constraints, including a lack of coronary angiography, hinder precise HF management in Cameroon [12]. The lack of recent data hinders the current updated understanding of HF sex-specific epidemiology, risk factors, and outcomes, preventing the development of targeted interventions in Cameroon´s resource-limited healthcare system. Analysing more recent data from hospitalized patients could reveal unique sex-specific HF patterns and guide tailored clinical strategies. This study therefore set out to investigate sex-specific patterns in patients hospitalised for HF at Laquintinie Hospital, Douala.

## Methods

This analytical cross-sectional study adheres to STROBE guidelines for transparent reporting [13].

**Study design and setting:** this was an analytical cross-sectional study at the cardiology unit of Laquintinie Hospital, a secondary-level facility in Douala, Cameroon, which has 70-80 patients admitted monthly, with 21-32 patients managed for heart failure (unpublished reports). Data covered admissions from January 2021 to December 2024.

**Participants:** eligible participants were patients of all ages hospitalised in the cardiology unit with a confirmed diagnosis of acute HF, defined by clinical symptoms (e.g., dyspnoea, fatigue, oedema), Framingham criteria, elevated natriuretic peptide levels (B-type natriuretic peptide (BNP) ≤100 pg/mL or N-terminal pro-BNP ≤300 pg/mL), and echocardiographic findings consistent with the 2021 ESC guidelines [14]. Inclusion criteria required at least one echocardiogram performed during hospitalisation to confirm aetiological type and/or permit classification of HF subtype: HF with reduced ejection fraction (HFrEF), mildly reduced ejection fraction (HFmrEF), or preserved ejection fraction (HFpEF). Exclusion criteria included patients with incomplete prescription or discharge outcome records or those diagnosed with cor pulmonale, pericardial disease, or congenital heart disease, as these conditions have distinct management protocols.

**Variables:** this study had four outcomes of interest (atrial fibrillation, ischemic cardiomyopathy, RAAS inhibitor use at discharge, and hypertension). Covariates were selected based on clinical relevance and statistical significance in our cohort: age, admission year, marital status, residence, systolic blood pressure, diabetes, renal disease (blood urea nitrogen value in mg/dL), and hypertension status (known and new-onset for the hypertension model). Hypertension was defined as a history of diagnosed hypertension or new-onset blood pressure ≥160/90 mmHg on admission, measured twice per ESC guidelines. Hypoxia was defined as oxygen saturation <90% on room air via pulse oximetry. Anemia was defined as haemoglobin <13 g/dL for males and <12 g/dL for females. ECG findings, including atrial fibrillation (irregular RR intervals, absent P waves) and ischemic changes (ST-segment deviations, Q waves), followed standard cardiology criteria [14-17] (McDonagh *et al*. WHO, Thygesen *et al*.).

Heart failure (HF) types were categorized using left ventricular ejection fraction (LVEF): HFrEF (LVEF ≤40%), HFmrEF (LVEF 41-49% with increased filling pressures), and HFpEF (LVEF ≥50% with evidence of diastolic dysfunction). Medication usage was documented prior to admission, during hospital stay, and at discharge, including diuretics, calcium channel blockers (CCBs), dobutamine, and digoxin.

**Sampling and sample size calculation:** a convenience sampling method was chosen due to practical considerations, given the anticipated challenges in retrieving comprehensive historical data. Using G*Power for a two-sample t-test comparing mean ages (men: 54.0 ± 16.9 years; women: 50.7 ± 19.5 years) in heart failure patients per Ogah *et al*. [11], assuming d = 0.25, α = 0.05, and 80% power, about 252 patients per group (504 total) were required, which translates to about 560 patients considering with 10% non-response rate. The sample size necessary for prediction models was based on the rule of thumb by Peduzzi *et al*. which recommends a minimum of 10 events per predictor variable to ensure adequate statistical power [18].

**Data sources and collection:** a structured tool captured: (1) demographics (age, sex, marital status, residence); (2) clinical characteristics (e.g., ejection fraction: HFrEF <40%, HFmrEF 40-49%, HFpEF ≥50%; systolic blood pressure; oxygen saturation); (3) laboratory findings (e.g., haemoglobin, blood urea nitrogen [BUN]); (4) medications (e.g., RAAS inhibitors); and (5) outcomes (mortality, hospital stay).

**Statistical analysis:** analyses used R (v4.4.1). The primary research question was: to what extent do sex-specific differences exist in clinical profiles, aetiologies, and in-hospital outcomes among hospitalized HF patients in this setting? Data included sociodemographic, clinical, laboratory, and outcome variables from all eligible patients. Descriptive statistics reported medians (IQR) for non-normal continuous variables (assessed via the Shapiro-Wilk test) and frequencies (%) with 95% CIs for categorical variables. Sex differences were tested using Chi-square or Fisher's exact tests for categorical variables, independent t-tests for normally distributed continuous variables, or Mann-Whitney U tests for non-normal continuous variables (p<0.05). Missing data (10.4% haemoglobin, 46.8% BUN, 5.2% blood pressure, 53.5% ECG) were handled using Multiple Imputation by Chained Equations (MICE, m=5, 10 iterations). Missingness was assumed to be missing at random (MAR) based on exploratory analyses showing correlations with observable variables (e.g., admission year, demographics) but no evidence of systematic bias tied to unobservable factors (e.g., no indication of missing not at random [MNAR] mechanisms). Sensitivity analyses assessed imputation robustness. We used penalized logistic regression (brglm2 package) to address sparse data and separation.

Adjusted odds ratios (AORs) with 95% CIs were estimated. Models were validated using 500 bootstrap iterations to assess overfitting, with calibration via Hosmer-Lemeshow tests. Weighted regression addressed class imbalance (e.g., ischemic cardiomyopathy: 6.6%). Model fit was evaluated via the area under the curve (AUC). Sex-stratified analyses explored interactions (e.g., age*sex).

**Ethical considerations:** the Regional Human Health Research Ethics Committee for the Littoral (ref: 2024/CE/CRH-LITTORAL) approved the study, waiving informed consent due to its retrospective design. Data were anonymized. The requirement for informed consent was waived due to the retrospective nature of the study. All procedures were carried out in accordance with applicable guidelines and regulations for the use of anonymized secondary data.

## Results

**Participants:** from January 2021 to December 2024, Laquintinie Hospital recorded 3,139 cardiology admissions, of which 757 patients (369 males (48.7%), 388 females (51.3%)) were included in the study, with no exclusions. No patients were lost to follow-up, given the focus on in-hospital outcomes. ECG data were available for 352 patients (46.5%) to diagnose ischemic cardiomyopathy and atrial fibrillation, while discharge medication records were complete for all patients.

**Descriptive data:** median age was 63 years (interquartile range (IQR): 51-74), with females older (66 years (IQR: 55-76) vs. 60 years (IQR: 49-71), p<0.001). Males were more likely married (63.4% (95% CI: 58.4-68.2) vs. 32.5% (95% CI: 28.0-37.3), p<0.001), while females were more often widowed (39.4% (95% CI: 34.6-44.4) vs. 5.7% (95% CI: 3.6-8.8), p<0.001). Admissions were stable across 2021-2024 (p-trend=0.184) as shown in Table 1. Comorbidities included chronic HF (34.2%, p=0.702) and diabetes (16.9%, p=0.384), with no sex differences.

**Table 1:** sociodemographic characteristics and sex-specific distribution in patients hospitalised for heart failure at Laquintinie Hospital, Douala, from 2021-2024

Sociodemographic variables	N (%)	Males n=369 (%)	Females n= 388 (%)	Crude OR (95%CI)^a^	p-value
**Year of admission**					
2021	136 (18.0)	59 (16.0)	77 (19.8)	-	-
2022	204 (26.9)	109 (29.5)	95 (24.5)	-	-
2023	184 (24.3)	86 (23.3)	98 (25.3)	-	-
2024	233 (30.8)	115 (31.2)	233 (30.8)	-	0.668*
Median age in years (Q1-Q3)	63.0 (51-74)	60.0 (49-71)	66.0 (55-76)	1.02 (1.01-1.03)	<0.001
Marital status					
Married	360 (47.6)	234 (63.4)	126 (32.5)	0.28 (0.21-0.37)	<0.001
Widow (er)	174 (23.0)	21 (5.7)	153 (39.4)	0.09 (0.06-0.15)	<0.001

COR^a^: crude odds ratio with males as the reference category

Males had higher tuberculosis history (6.8% (95% CI: 4.6-10.0) vs. 2.6% (95% CI: 1.4-4.8), p=0.009) and gout (1.9% vs. 0%, p=0.007). Males were less likely to use cardiovascular medications prior to admission (73.7% (95% CI: 69.0-78.0) vs. 57.7% (95% CI: 52.8-62.5), p<0.001). Females used more diuretics (18.8% vs. 11.4%, p=0.005) and renin-angiotensin-aldosterone system (RAAS) inhibitors (16.8% vs. 8.9%, p=0.002) as shown in Table 2.

**Table 2:** comorbidities and medications of heart failure patients hospitalised for heart failure at Laquintinie Hospital, Douala, from 2021-2024

Past medical history	N (%)	Males n=369 (%)	Females n= 388 (%)	COR (95% CI)^a^	p-value
**Comorbidities**					
Diabetes	128 (16.9)	67 (18.2)	61 (15.7)	0.84 (0.57-1.23)	0.384
Hypertension	432 (57.1)	182 (49.3)	250 (64.4)	1.86 (1.39-2.49)	< 0.001
Known hypertension	210 (48.6)	88 (48.4)	122 (48.8)		
New-onset hypertension	222 (51.4)	94 (41.6)	128 (51.2)		
History of TB	36 (4.6)	25 (6.8)	10 (2.6)	0.36 (0.17-0.77)	0.009
History of lung pathology^b^	65 (8.6)	39 (10.6)	26 (6.7)	0.61 (0.36-1.02)	0.069
**Routine CV medications**					
**SGLT2 inhibitors**					
Calcium channel blockers	125 (16.5)	42 (11.4)	83 (21.4)	0.47 (0.32-0.71)	<0.001
Diuretics	115 (15.2)	42 (11.4)	73 (18.8)	1.8 (1.20-2.72)	0.005
ACEI	98 (12.9)	33 (8.9)	65 (16.8)	2.05 (1.31-3.20)	0.002
ARAII	29 (3.8)	11 (3.0)	18 (4.6)	1.58 (0.74-3.40)	0.260
Beta blockers	90 (11.9)	35 (9.5)	55 (14.2)	1.58 (1.00-2.47)	0.048
No routine CV medication	496 (65.5)	272 (73.7)	224 (57.7)	1.31 (1.14-1.50)	<0.001

ACEI: angiotensin converting enzyme inhibitors; ARAII: angiotensin II receptor antagonists; COPD: chronic obstructive pulmonary disease; CV: cardiovascular; CVA: cerebrovascular accident; MRA: mineralocorticoid receptor antagonist; TB: tuberculosis; ^b^: history of lung pathology (asthma, pulmonary embolism, COPD, TB); COR^a^: crude odds ratio with males as the reference category; SGLT2: sodium-glucose cotransporter 2

Clinical findings showed females had lower oxygen saturation (93% (IQR: 88-97) vs. 95% (IQR: 91-97), p=0.021) and higher hypoxia (oxygen saturation <90% on room air: 33.6% (95% CI: 29.0-38.5) vs. 21.7% (95% CI: 17.7-26.3), p=0.001). Males had more ischemic changes (ST-segment deviations, Q waves: 16.4% (95% CI: 11.4-22.9) vs. 7.3% (95% CI: 4.1-12.5), p=0.012) and ischemic cardiomyopathy (8.7% (95% CI: 6.2-12.1) vs. 4.9% (95% CI: 3.1-7.6), p=0.041). HF with reduced ejection fraction (HFrEF, <40%) predominated (54.9%), with no sex differences (p=0.161). No coronary angiography was performed.

Males had higher BUN (>60 mg/dL: 43.8% (95% CI: 37.4-50.4) vs. 30.1% (95% CI: 24.5-36.4), p=0.003) and renal disease as an HF precipitant (7.6% vs. 3.6%, p=0.019). Anemia, defined as haemoglobin <13 g/dL for males and <12 g/dL for females, was present in 66.8% (304/455) of patients with available haemoglobin measurements. The prevalence was 62.9% in males (144/229) and 70.8% in females (160/226). Females had lower odds of anemia (OR 0.77, 95%CI: 0.58-1.03, p=0.080) as shown in Table 3.

**Table 3:** admission characteristics (clinical, diagnostic, and laboratory findings) of patients hospitalised for heart failure at Laquintinie Hospital, Douala, from 2021-2024

Admission characteristics	N	Variable distribution	Males n=369 (%)	Females n= 388 (%)	COR (95% CI)^a^	p-value
**Clinical characteristics**						
Median SBP (IQR; Q1-Q3)	753	137 (114-162)	138 (113-168)	136 (116-160)	0.99 (0.995-1.003)	0.713
Hypotension (SBP <90mmHg)	753	35 (4.6)	13 (6.3)	12 (3.1)	0.48 (0.24-0.98)	0.044
Median SaO2% (IQR; Q1-Q3)	679	94 (89-97)	95 (91-97)	93 (88-97)	0.98 (0.96-1.00)	0.021
Hypoxia (SaO2% <90%)	679	189 (27.8)	72 (21.7)	117 (33.6)	1.78 (1.27-2.49)	0.001
**Diagnostic investigations**						
ECG done	757	352 (46.5)	173 (46.9)	179 (46.1)	1.03 (0.77-1.37)	0.884
Atrial fibrillation	352	94 (26.7)	33 (19.1)	61 (34.1)	1.89 (1.21-2.97)	0.002
Ischemic changes	352	41 (11.7)	28 (16.4)	13 (7.3)	0.42 (0.21-0.83)	0.012
Echocardiography done	757	412 (54.4)	207 (56.1)	205 (52.8)	0.88 (0.66-1.17)	0.382
HFpEF	412	152 (36.9)	72 (34.8)	80 (39.0)	1.07 (0.75-1.53)	0.704
HFmrEF	412	42 (10.2)	20 (9.7)	22 (10.7)	1.05 (0.56-1.96)	0.881
HFrEF	412	218 (54.9)	115 (55.6)	103 (50.2)	0.80 (0.58-1.09)	0.161
Median EF (IQR; Q1-Q3)	412	39 (26-60)	38 (35-60)	40 (30-60)	1.01 (1.00-1.02)	0.134
**Laboratory findings**						
Median BUN in mg/dL (IQR; Q1-Q3)	403	49 (32.5-77.9)	55 (37-86)	42 (29-72)	0.99 (0.99-1)	0.003
BUN>60 mg/dL	403	149 (37.2)	91 (43.8)	58 (30.1)	0.54 (0.37-0.77)	0.001
Haemoglobin level	455	11.3 (9.6-12.9)	11.4 (9.8-13.4)	11.2 (9.4-12.6)	0.94 (0.87-1.00)	0.060
Anaemia	455	304 (66.8)	144 (62.88)	160 (70.8)	0.77 (0.58-1.03)	0.080

COR^a^: crude odds ratio with males as reference category; detailed characteristics can be found in the supplementary materials; SBP: systolic blood pressure; HFpEF: heart failure with preserved ejection fraction; HFmrEF: heart failure mildly reduced ejection fraction; HFrEF: heart failure with reduced ejection fraction; EF: ejection fraction; BUN: blood urea nitrogen

Hypertensive heart disease was the leading aetiology (28.5%), more common in females (p<0.001). Females had more arrhythmic cardiomyopathy (12.9% (95% CI: 9.8-16.8) vs. 6.5% (95% CI: 4.3-9.7), p=0.004) and peripartum cardiomyopathy (3.1% vs. 0%, p=0.001). Non-identified aetiologies accounted for 14.8%, as shown in Table 4.

**Table 4:** precipitating factors and aetiologies of heart failure in patients hospitalised at Laquintinie Hospital, Douala, from 2021-2024

Aetiologies and precipitating factors	N	Variable distribution	Males n=369 (%)	Females n= 388 (%)	COR (95% CI)^a^	p-value
**Precipitating factors**						
Infection	757	120 (15.9)	61 (16.5)	59 (15.2)	0.91 (0.61-1.34)	0.618
Pneumonia	757	26 (3.4)	17 (4.6)	9 (2.3)	0.49 (0.22-1.12)	0.090
Lifestyle non-adherence	757	22 (2.9)	10 (2.7)	12 (3.1)	1.15 (0.49-2.68)	0.754
Renal disease	757	42 (5.5)	28 (7.6)	14 (3.6)	0.46 (0.24-0.88)	0.019
No precipitating factor identified	757	341 (45.0)	161 (43.6)	180 (46.4)	1.12 (0.84-1.49)	0.446
**Aetiologies**						
Ischemic cardiomyopathy	757	51 (6.7)	32 (8.7)	19 (4.9)	0.54 (0.30-0.97)	0.041
Arrhythmic cardiomyopathy	757	74 (9.8)	24 (6.5)	50 (12.9)	2.13 (1.28-3.54)	0.004
Dilated cardiomyopathy	757	98 (12.9)	57 (15.4)	41 (10.6)	0.65 (0.42-0.99)	0.047
Hypertensive heart disease	757	216 (28.5)	111 (30.1)	105 (27.1)	0.86 (0.63-1.18)	0.358
Pericardial disease	757	22 (2.9)	16 (4.3)	6 (1.5)	0.35 (0.13-0.90)	0.029
Peripartum cardiomyopathy	757	12 (1.6)	-	12 (3.1)	-	-

COR^a^: crude odds ratio with males as reference category

**Outcome data:** females were more likely to receive RAAS inhibitors at discharge (33.5% (95% CI: 28.9-38.4) vs. 26.3% (95% CI: 22.0-31.1), p=0.031). Females were more likely to have atrial fibrillation (34.1% (95% CI: 27.3-41.6) vs. 19.1% (95% CI: 13.7-26.0), p=0.002). but less ischaemia on ECG than males (7.3% vs 16.4%, p=0,021). Hypertension was prevalent in 432 patients (57.1%, 95% CI: 53.5-60.6), with 210 (48.6%) having known hypertension and 222 (51.4%) new-onset; females had higher rates (250/388 (64.4%, 95% CI: 59.5-69.1) vs. 182/369 (49.3%, 95% CI: 44.2-54.4), p<0.001). Median hospital stay was 8 days (IQR: 5-12), with no sex difference (p=0.190). In-hospital mortality was 17.0% (95% CI: 14.4-20.0), with no sex difference (p=0.288) (Table 5).

**Table 5:** management and outcomes of patients hospitalised for heart failure at Laquintinie Hospital, Douala, from 2021-2024

Management and outcomes	Variable distribution	Males n=369 (%)	Females n= 388 (%)	COR (95% CI)^a^	p-value
**Medications during hospitalisation**					
Dobutamine	39 (5.2)	23 (6.2)	16 (4.1)	0.65 (0.34-1.25)	0.192
Beta blockers	299 (39.5)	154 (41.7)	145 (37.4)	0.83 (0.62-1.12)	0.220
RAAS inhibitors	231 (30.5)	100 (27.1)	131 (33.8)	1.37 (1.00-1.87)	0.049
**Medications at discharge**					
Calcium channel blockers	163 (21.5)	77 (20.9)	86 (22.2)	1.08 (0.76-1.53)	0.724
Loop diuretics	490 (64.7)	233 (63.1)	257 (66.2)	1.15 (0.85-1.54)	0.373
Beta blockers	337 (44.5)	163 (44.2)	174 (44.8)	1.03 (0.77-1.37)	0.852
Mineralocorticoid receptor antagonists	170 (22.5)	83 (22.5)	97 (22.4)	1.00 (0.71-1.40)	0.981
RAAS inhibitors	227 (30.0)	97 (26.3)	130 (33.5)	1.41 (1.03-1.93)	0.031
SGLT2 inhibitors	83 (11.0)	40 (10.8)	43 (11.1)	1.03 (0.65-1.62)	0.915
Median duration of hospitalisation in days (IQR; Q1-Q3)	8 (5-12)	7 (5-11)	8 (5-12)	1.01 (1.00-1.02)	0.190
Discharged death	129 (17.0)	57 (15.4)	72 (18.6)	1.25 (0.85-1.83)	0.288

RAAS: renin-angiotensin aldosterone system; SGLT2: sodium-glucose cotransporter 2; COR^a^: crude odds ratio with males as reference category

**Figure 1:**
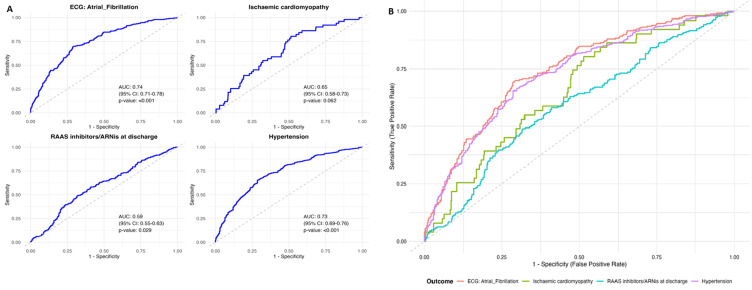
A, B) model performance for sex-specific heart failure outcomes, 2021-2024 (receiver operating characteristic (ROC) curves for penalized logistic regression models predicting atrial fibrillation (AUC=0.742, 95% CI: 0.705-0.779), ischemic cardiomyopathy, renin-angiotensin-aldosterone system inhibitor use, and hypertension, adjusted for age, admission year, marital status, residence, systolic blood pressure, diabetes, renal disease, and hypertension status)

**Regression analysis:** penalized logistic regression models, adjusted for age, admission year, marital status, residence, systolic blood pressure, diabetes, renal disease (BUN >60 mg/dL), and hypertension status, showed females had higher odds of atrial fibrillation (adjusted odds ratio (AOR)=3.64, 95% CI: 1.91-6.94, p<0.001) and hypertension (AOR=1.95, 95% CI: 1.38-2.76, p<0.001), and lower odds of ischemic cardiomyopathy (AOR=0.48, 95% CI: 0.25-0.95, p=0.035). RAAS inhibitor use at discharge showed no significant sex difference (AOR=1.41, 95% CI: 0.99-2.00, p=0.052). The atrial fibrillation model had strong discrimination (area under the curve (AUC)=0.742, 95% CI: 0.705-0.779) (Figure 1). Sensitivity analyses confirmed imputation robustness for missing data. The Hosmer-Lemeshow Goodness-of-Fit test indicated that the logistic regression models for atrial fibrillation, hypertension, and ischemic cardiomyopathy demonstrated good fit (p>0.05 for all), suggesting their predictions align well with observed outcomes. Conversely, the model for RAAS Inhibitors/ARNI at discharge showed a poor fit (p=0.047), indicating a significant discrepancy between its predictions and observed data.

## Discussion

This study analysed 757 patients (369 males, 388 females) hospitalized for heart failure (HF) at Laquintinie Hospital, Douala, from 2021-2024. Females were older (median: 66 vs. 60 years, p<0.001), more likely to be widowed (39.4% vs. 5.7%), and less likely to be married (32.5% vs. 63.4%). Hypertension was more prevalent in females (56.7% vs. 40.1%), while males had a higher history of tuberculosis (6.8% vs. 2.6%, p=0.009). Females were more likely to receive calcium channel blockers, diuretics, ACE inhibitors, and beta-blockers (all p<0.05). Clinically, females had higher hypoxia rates (33.6% vs. 21.7%), while males had more renal disease as a precipitating factor (7.6% vs. 3.6%). Females had more atrial fibrillation (34.1% vs. 19.1%, p=0.002), and males more ischaemia on ECG (16.4% vs. 7.3%). Aetiologically, females had less ischemic (4.9% vs. 8.7%, p=0.041) and dilated cardiomyopathy (10.6% vs. 15.4%), but more arrhythmic cardiomyopathy (12.9% vs. 6.5%). Females received more RAAS inhibitors during hospitalization and discharge (p<0.05). Mortality was 17.0%, with no sex differences.

In this study, females were older than men. This contrasts with the study from Ogah *et al*. using a cohort of sub-Saharan African patients, where males were older [11]. In western studies, women with HF are typically older, due to higher prevalence of HFpEF and longer life expectancy [19] as shown by this study (HFpEF; 34.8% vs 39.0%), although without statistical significance. Age was a modulating factor for atrial fibrillation (AF), as expected with structural and functional atrial anion changes associated with ageing [20]. For this reason, it is not surprising that women, who were older, also had higher rates of AF. Hypertension was also commoner in females (56.7% vs. 40.1%) in this study, correlating with the higher rates of AF, as hypertension is recognised as the most common cause of AF, with risks increased by more than 30% at any given age [21]. However, hypertension was influenced by the comorbid diagnosis of diabetes and routine use of ACEI and CCBs, eliminating the initial gender-specific association. Since the female gender retained higher odds of AF irrespective of age, year of admission, marital status, hypertension, chronic heart failure, hypoxia, and beta-blockers, other mechanisms may be at play.

Women have a greater burden of atrial fibrosis compared to men [22,23], which may predispose them to more complications from AF such as HF [24]. Studies have revealed that women have a greater population with AF but are less likely to receive anticoagulation and have less time in therapeutic range (TTR) when on warfarin for AF compared to men [24]. The higher prevalence of widowhood among females (39.4% vs. 5.7%, p<0.001) and higher married rates among males (63.4% vs. 32.5%, p<0.001), in addition to older age in women, may therefore be a reflection of sex-specific socioeconomic disparities in healthcare access and late diagnosis [25], unexplored in this current study. Longitudinal studies should be conducted to explore the effect of gender and socioeconomic status on diagnosis and outcomes of HF in sub-Saharan Africa.

Although pulmonary comorbidities (10.6% vs 6.7%; p=0.069) and history of TB (6.8 vs 2.6; p=0.009) were more common in men, more females presented with hypoxia (33.6% vs 21.7%, p=0.001). Some authors have reported that elderly women exhibit greater hypoxemia and reduced chemoreflex sensitivity during hypoxia compared to men, potentially impairing oxygen extraction despite increased haemoglobin oxygen dissociation [26]. Moreover, anaemia, a known correlate to hypoxia [27,28], was more common in women (62.9% vs 57.1%, p=0.537).

The study also found a higher hypotension rate in males (6.3% vs. 3.1%, p=0.004), consistent with the greater prevalence of HFrEF (55.6% vs. 50.2%, p=0.161) and ischaemia (16.4% vs. 7.3%), where hypotension is a frequent complication [29,30]. Although no sex-specific differences were noted in intrahospital outcome, previous authors have reported a higher 30-day mortality risk (7.1% vs. 2.9%) associated with intrahospital hypotension [31]. This highlights the need for more post-discharge longitudinal studies in sub-Saharan Africa. Ischemic heart disease (IHD) occurs more commonly in males and is driven by factors such as older age, diabetes, and hypertension [32,33]. However, amongst these risk factors, only diabetes was more common in males (18.2% vs 15.7%, p= 0.384), but was not an independent predictor of IHD. In this study, 63.4% of women were ≥60 years, an age group with a higher incidence of ischemic heart disease (IHD) compared to age-matched men, likely due to post-menopausal low oestrogen levels contributing to atherosclerotic plaque complications [34,35]. As no patient did a coronary angiography, the risk of misclassification of IHD is high in this study (14.8% idiopathic causes), more so as classical symptoms of ischemia like chest pain occur less commonly in women (31% versus 42%) [34,36]. It is concerning that ischemic heart disease (IHD) in women may be misattributed to menopause or emotional stress, resulting in delayed diagnosis, undertreatment, and fewer angiography referrals, despite younger women often presenting with worse baseline risk factors [37]. The lack of gender differences in echocardiography use (56.1% males vs. 52.8% females, p=0.382) may imply access to diagnostic investigations is not influenced by gender. Further studies are required to ensure adequate IHD exploration in sub-Saharan Africa.

Renin-angiotensin-aldosterone system (RAAS) inhibitor during hospitalization (33.8% vs. 27.1%, p=0.049) and at discharge (33.5% vs. 26.3%, p=0.032) was more common in women. National Institute for Health and Care Excellence (NICE) advises against initiating RAAS inhibitors in CKD patients with a baseline serum potassium level above 5.0 mEq/L and recommends stopping them if serum potassium reaches or exceeds 6.0 mmol/L. End-stage kidney disease (ESRD) occurs 50% more often in men, despite chronic kidney disease (CKD) being more common in women [38], as confirmed in this study, where males were more likely to have renal disease as a precipitating factor (7.6% vs. 3.6%). This was further correlated with their higher rates of elevated blood urea nitrogen (>60 mg/dL, 43.8% vs. 30.1%, p=0.005) and hyperkalaemia (14.4% vs 7.2%, p=0.051). Because renal disease highlights target organ damage and advanced complications, possibly from chronic hypertension, diabetes, or reduced cardiac output (HFrEF) [39], it was surprising to note that males were more likely to use no cardiovascular medications (73.7% vs. 57.7%, p<0.001) at presentation. The slightly higher medication non-adherence as a precipitating factor of HF (11.4% vs 10.8%; p=0.807) further supported this finding, which potentially reflects lower healthcare-seeking behaviour in African men [40]. Future studies should explore the potential effect of routine renal function screening and healthcare-seeking behaviour interventions on the incidence of HF hospitalisation in African settings.

Similar to reports by Ogah *et al*. [11], the mortality among heart failure patients did not display sex-specific variations between male and female groups, even though there had been sex-specific differences within clinical characteristics like higher rates of hypoxia among females and higher prevalence of renal illness and hypotension among males. This lack of variation can be attributed to offsetting risks, where the higher prevalence of heart failure with preserved ejection fraction (HFpEF) as well as atrial fibrillation (AF) among females, combined with the higher heart failure with reduced ejection fraction (HFrEF) burden and hypotension but younger age among males, produces similar mortality risks caused by different though equivalent severity of pathophysiological mechanisms. In addition, the high prevalence of cardiovascular drug non-use among males could neutralize any potential benefits of their lower prevalence of hypoxia, while females' older age group could counter their comparatively less severe renal impairment. These findings suggest that while sex-specific factors influence heart failure presentation, their effect on mortality could be offset by level risk profiles, indicating the need for sex-specific individualized management strategies that target underlying comorbidities as well as improving therapy for both sexes. However, the poor model fit for RAAS inhibitors (Hosmer-Lemeshow p=0.047) indicates that associations with this outcome should be interpreted cautiously, as the model may not fully capture the relationships; these findings are preliminary and require validation.

**Limitations and strengths:** this study provides substantial insight into sex-specific heart failure (HF) features within a sub-Saharan Africa cohort, though not without some limitations. Convenience sampling of only hospitalized cases introduces selection bias (a form of referral bias), likely representing more severe HF cases and overestimating in-hospital mortality (17.0%) compared to the general community. Omission of coronary angiography and high prevalence of idiopathic diagnoses (14.8%) potentially caused misclassification of ischemic heart disease (IHD), especially in females, where symptoms are less prominent; this may distort the observed sex differences in ischemic cardiomyopathy (AOR=0.48), masking true associations. The high missing rate for ECG data (53.5%) affects the robustness of atrial fibrillation and ischemic cardiomyopathy models, potentially biasing results toward patients sick enough to receive an ECG. Inclusion of 2021 in the analysis, a period when COVID-19 was still prevalent, could have led to underestimation of HF mortality, as this period was associated with poorer health access. Despite such drawbacks, the strengths of the study reside within its massive sample size (n=757) and thorough clinical profiling, including a broad range of predictors (i.e., hypoxia, renal disease, medication) within a setting where HF information is scarce, thus allowing for a tangible base for future research studies.

**Implications for policy, practice, and research:** the findings highlight the need for sex-specific HF management in sub-Saharan Africa. Policy should prioritize routine renal function screening and cardiovascular medication adherence programs, particularly for males. In practice, clinicians should focus on early hypoxia detection while addressing hypotension in males with cautious use of guideline-directed medical therapy (GDMT). Research should investigate longitudinal outcomes, IHD misclassification risks, especially in females, and socioeconomic barriers (e.g., widowhood, healthcare-seeking behaviour) to optimize HF care and reduce mortality in this population.

## Conclusion

This study highlights distinct sex-specific HF profiles in a sub-Saharan African setting, with females exhibiting older age, higher hypoxia, and atrial fibrillation, and males showing greater renal disease, hypotension, and medication non-use, yet no mortality d ifference (17.0%). These findings reflect balanced risk profiles driven by HFpEF in females and HFrEF in males, emphasizing the need for tailored interventions to address comorbidities and treatment gaps. By informing targeted policies and practices, this research paves the way for improved HF management and equitable care in resource-limited settings. However, due to limitations like missing data and potential biases, findings on RAAS inhibitors are preliminary and require cautious interpretation.

### What is known about this topic

- Heart failure affects 55.5 million people globally, with sub-Saharan Africa experiencing a high in-hospital mortality rate of 26.4%, in Cameroon, hypertension is a critical risk factor for HF, with a high prevalence and significant new-onset cases, particularly in females, potentially highlighting gaps in primary care;
- Females often present with hypertension-driven HF with preserved ejection fraction (HFpEF), while males are more likely to have ischemic-driven HF with reduced ejection fraction (HFrEF); risk factors such as coronary heart disease, diabetes, and others differ by sex, influencing HF incidence and outcomes;
- Previous studies, like the THESUS-HF study (2007-2010), noted higher atrial fibrillation in females and worse renal markers in males, but recent data on sex-specific HF epidemiology in Cameroon are limited.

### What this study adds

- The study confirms females are older, have higher rates of hypertension (64.4% vs. 49.3%), atrial fibrillation (AOR=3.64), and arrhythmic cardiomyopathy, and receive more RAAS inhibitors at discharge (33.5% vs. 26.3%); males exhibit more ischemic cardiomyopathy (AOR=0.48), renal dysfunction (43.8% vs. 30.1%), and lower cardiovascular medication use prior to admission (73.7% vs. 57.7%);
- Despite sex-specific differences in clinical characteristics (e.g., higher hypoxia in females, more hypotension in males), in-hospital mortality (17.0%) showed no sex difference, suggesting balanced risk profiles potentially due to offsetting factors like HFpEF in females and HFrEF in males;
- The findings highlight the need for targeted HF management strategies in sub-Saharan Africa, such as atrial fibrillation screening for females, renal function monitoring for males, and addressing medication non-adherence, particularly in males, to optimize care in resource-limited settings.
